# The functional networks of a novel environment: Neural activity mapping in awake unrestrained rats using positron emission tomography

**DOI:** 10.1002/brb3.1646

**Published:** 2020-06-20

**Authors:** Matthew McGregor, Kaleigh Richer, Mala Ananth, Panayotis K. Thanos

**Affiliations:** ^1^ Behavioral Neuropharmacology and Neuroimaging Laboratory on Addictions Clinical Research Institute on Addictions Department of Pharmacology and Toxicology Jacobs School of Medicine and Biosciences State University of New York at Buffalo Buffalo NY USA; ^2^ Department of Psychology State University of New York at Buffalo Buffalo NY USA; ^3^ Department of Neurobiology State University of New York at Stony Brook Stony Brook NY USA

**Keywords:** brain glucose metabolism, FDG, functional connectivity, imaging, novel environment, olfaction

## Abstract

**Introduction:**

Novel environment stimulation is thought to have an important role in cognitive development and has been shown to encourage exploratory behavior in rats. However, psychopathology or perceived danger or stress can impede this exploratory drive. The balance between brain circuits controlling the exploratory drive elicited by a novel environment, and the avoidance response to stressors, is not well understood.

**Methods:**

Using positron emission tomography (PET) and the glucose analog [^18^F]fluorodeoxyglucose (18F‐FDG), we assessed awake brain glucose metabolism (BGluM) in rats while in a novel environment (cage of an unfamiliar male rat) and non‐novel environment (the animal's home cage).

**Results:**

Exposure to the novel environment increased BGluM in regions associated with vision (visual cortex), motor function and motivated behavior (striatum and motor cortex), and anxiety (stria terminalis), and decreased BGluM in regions associated with auditory processing (auditory cortex, insular cortex, inferior colliculus), locomotor activity (globus pallidus, striatum, motor cortex, ventral thalamic nucleus), spatial navigation (retrosplenial cortex), and working memory (hippocampus, cingulate cortex, prelimbic cortex, orbitofrontal cortex).

**Conclusion:**

These results suggest that the novel cage is a stressful environment that inhibits activity in brain regions associated with exploratory behavior. Patterns of inhibition in the novel cage also support the proposed rat default mode network, indicating that animals are more cognitively engaged in this environment. Additionally, these data support the unique capability of combining FDG‐PET with psychopharmacology experiments to examine novelty seeking and brain activation in the context of decision making, risk taking, and cognitive function more generally, along with response to environmental or stress challenges.

## Graphical abstract

The widespread inhibition of brain activity in regions associated with exploratory behavior suggest that the novel cage is a stressful environment, a result which supports the elevated corticosterone levels in a novel environment reported by other groups. Additionally, patterns of inhibition in the novel environment are consistent with proposed rat Default Mode Network models, indicating that the animals were more cognitively engaged in this environment. Our results demonstrate the capability of FDG PET to identify cognitive changes in response to novelty and stress.

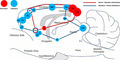

## 1 INTRODUCTION

There is significant evidence to suggest that novelty can encourage exploratory behavior. Novelty seeking is a personality trait that can be described by exploratory activity wherein individuals look for novel and exciting stimulation and respond strongly to the spike in dopamine release in the brain when they experience something novel (Cloninger, Svrakic, & Przybeck, 1993). In fact, studies have identified numerous genes and biochemical changes involving the noradrenergic (Garvey, Noyes, Cook, & Blum, 1996;Sara, Dyon‐Laurent, & Herve, 1995) and dopamine system (Benjamin et al., 1996;Ebstein & Belmaker, 1997) that are correlates of novelty seeking. Furthermore, high novelty seeking is considered to be a vulnerable trait that is associated with predicting risky behaviors like alcohol (Wellman, Contreras, Dugas, O'Loughlin, & O'Loughlin, 2014;Wills, Windle, & Cleary, 1998) and substance use dependency (Foulds, Boden, Newton‐Howes, Mulder, & Horwood, 2017), gambling (Cunningham‐Williams et al., 2005), ADHD (Downey, Stelson, Pomerleau, & Giordani, 1997), and binge eating disorder (Grucza, Przybeck, & Cloninger, 2007). Exploration in response to novelty is seen in animals as well. Rats spend more time exploring a novel object than a familiar one (Ennaceur, 2010) and show a preference for novel environments over familiar ones (Hall, Humby, Wilkinson, & Robbins, 1997). Yet several factors can interfere with this novelty seeking or exploratory drive. Novel objects or environments sometimes elicit an avoidance reaction in rats if the stimulus is perceived as dangerous, or if confinement to the novel environment induces stress (Bevins et al., 2002; Bind, Minney, Rosenfeld, & Hallock, 2013). The interplay between functional brain circuits controlling the exploratory drive elicited by a novel stimulus, and the avoidance response to stressors, is not well understood.

Exploratory behavior in rats is characterized by increased locomotion and rearing, while anxiety is characterized by immobility and self‐grooming (Cirulli, De Acetis, & Alleva, 1998). Olfaction is also heavily involved in exploration, as rats will spend more time sniffing a novel object than a familiar one (Ennaceur & Delacour, 1988). The novel cage test is an accepted method for assessing these behaviors. Rats are placed in a clean, novel cage, and incidence of exploratory and anxiety behaviors is recorded during a given time interval (Marques, Olsson, Ogren, & Dahlborn, 2008). By examining differences in exploratory behavior (olfactory activity, rearing, locomotion) and risk assessment (stretched attend posture, time spent in specified regions), characteristics of the animals can be considered. Results tend to correlate with behavior in elevated plus maze, open field, concentric square field, and rat exposure tests, which are also used for assessing anxiety and exploratory behavior.

The response to a novel stimulus is indeed influenced by the environment, although seemingly contradictory conclusions have been made regarding this relationship. One group found that rats will spend more time exploring a novel object when in a familiar environment (Bevins et al., 2002). This is contradicted by other findings that show decreased novel object exploration when the animal was familiar with the environment, although this may be because the novel environment presents more novel areas to explore (Powell, Geyer, Gallagher, & Paulus, 2004). Novel environments have been shown to induce moderately elevated corticosterone levels in rats, indicating this environment is a mild stressor (Brown, Uhlir, Seggie, Schally, & Kastin, 1974). The behavioral response to stress may differ between subjects. In some, known as high‐responding rats, exposure to the novel environment resulted in high rates of exploratory behavior not seen in a familiar environment, yet corticosterone levels were still elevated (Kabbaj, Devine, Savage, & Akil, 2000). Low responding rats exhibited less exploratory behavior, along with elevated corticosterone levels in the novel environment.

The presence of a conspecific has demonstrably attenuated the stress response in rats. In both periadolescent and adult male rats, the presence of another male rat while in a novel environment decreased circulating corticosterone levels (Terranova, Cirulli, & Laviola, 1999). This is true whether the paired rat is familiar or unfamiliar to the test rat. The presence of an unfamiliar male rat during a conditioned fear stimulus test also lowered corticosterone levels and reduced freezing behavior as compared to rats exposed to the conditioned stimulus alone (Kiyokawa, Hiroshima, Takeuchi, & Mori, 2014). This social buffering effect holds even when the same experiment is performed in the cage of a conspecific without the other animal present, indicating that olfactory cues can mediate the stress response (Kiyokawa, Honda, Takeuchi, & Mori, 2014). This effect is greater in the cage of a familiar than an unfamiliar conspecific, but both attenuate stress behavior compared to rats placed in a clean novel cage. Stress can also be induced in rats via odors secreted by conspecifics in stressful situations. Male rats release an alarm pheromone when stressed, which alters behavior in other rats by increasing anxiety and defensive behaviors (Kiyokawa, Kikusui, Takeuchi, & Mori, 2007). Preference for the compartment containing odors of another rat decreased when the other rat was subjected to a stressful foot shock condition; however, the stress odor also increased locomotor activity (Mackay‐Sim & Laing, 1980).

There has been significant interest in regard to the brain activity underlying these behaviors. Expression of the protein c‐Fos is often measured as an indirect cellular indicator of brain activity, as its expression correlates with neuronal firing. Rats that explored a novel olfactory cue training apparatus showed increased c‐Fos expression in the occipital cortex and superior colliculus (visual system), olfactory bulb and piriform cortex (olfactory system), and hippocampus, as compared to rats that were not exposed to the apparatus (Hess, Lynch, & Gall, 1995). In another c‐Fos expression study, exploration of a novel environment was found to activate the hippocampus and reward circuit (prelimbic cortex, ventral tegmental area, nucleus accumbens), while inhibiting amygdala activity (Bourgeois et al., 2012). The hippocampus is most frequently looked at in the context of learning in a novel environment. It is involved in both encoding and retrieving memories, and thereby is hypothesized to compare present stimuli with past experiences, directing attention to novel aspects of the current environment. There is an association between environmental novelty and c‐Fos expression in CA1 neurons of the hippocampus, layer five of the entorhinal cortex, and the perirhinal cortex (VanElzakker, Fevurly, Breindel, & Spencer, 2008). However, c‐Fos expression in motor and sensory areas was not associated with the degree of exploratory behavior, indicating the limits of c‐Fos as a correlate of brain activity.

Functional imaging provides another opportunity to indirectly evaluate brain activity during exploratory behavior. Many studies have sought to examine the relationship between the reward pathway and novelty‐seeking behavior, with some groups isolating the effects of a novel stimulus. In a human fMRI study, the substantia nigra/ventral tegmental area was activated by novel stimuli, whether the presented novel stimulus was expected or not. This suggests that dopaminergic processing of novelty may drive a motivational exploratory signal. Dopaminergic modulation from the SN/VTA enhances hippocampal plasticity, encouraging memory formation in response to novelty (Wittmann, Bunzeck, Dolan, & Duzel, 2007). Reward‐independent novel cues have also been found to increase activation in the medial and lateral occipital cortex, fusiform gyrus, dorsal anterior cingulate cortex, and hippocampus, as well as inhibit activity in the superior parietal cortex, medial and lateral prefrontal cortex, in human fMRI studies (Krebs, Schott, Schutze, & Duzel, 2009). Another group noted activation in the inferior frontal gyrus, insula, tempo‐parietal junction, and anterior cingulate in response to a novel cue, with decreased activation in the prefrontal, medial, and inferior temporal regions in response to a repeated (non‐novel) cue (Ranganath & Rainer, 2003). Novelty‐seeking behavior is also dependent on the frontal cortex, with patients having injury to the frontal cortex being apathetic toward novel aspects of their environment. This disinterest in novelty correlates with event‐related potentials, specifically an alteration of the stimulus evaluation P3 wave in the frontal cortex (Daffner et al., 2000). This is backed by fMRI data showing increased activation of areas of the frontal cortex in response to novelty (Wittmann et al., 2007).

The behavioral response to novelty is highly variable and sensitive to internal and external factors. While the brain regions involved in novelty seeking and detection have been investigated with functional imaging in humans, there is a notable dearth of similar research in rats. Given the correlation between the novelty seeking trait and risky behaviors that affect human health, investigation of the brain response to novelty in the context of drug use, stress, and other factors is needed. This requires an animal model for behavioral experiments and an understanding of the brain activity underlying novelty detection and seeking in that model.

In the present study, we sought to identify regions of the rat brain that are activated by a novel environment (the cage of an unfamiliar male rat). Using positron emission tomography (PET) and the glucose analog [^18^F] fluorodeoxyglucose (18F‐FDG), we can quantify a correlate of awake neural activity in specific regions by measuring regional brain glucose metabolism (BGluM) in rodents (Michaelides et al., 2012; Rice, Saintvictor, Michaelides, Thanos, & Gatley, 2006; Thanos, Michaelides, Benveniste, Wang, & Volkow, 2008; Thanos et al., 2013, 2016). Measured uptake of 18F‐FDG using this method is a well‐established correlate of neural activity (Sokoloff, 1981). In quantifying the changes in BGluM in rats exposed to a novel environment, we demonstrate the capability of this method for measuring brain activation in response to novelty with a high degree of specificity.

## 2 METHODS

### 2.1 Experimental procedure

**Figure 1:**
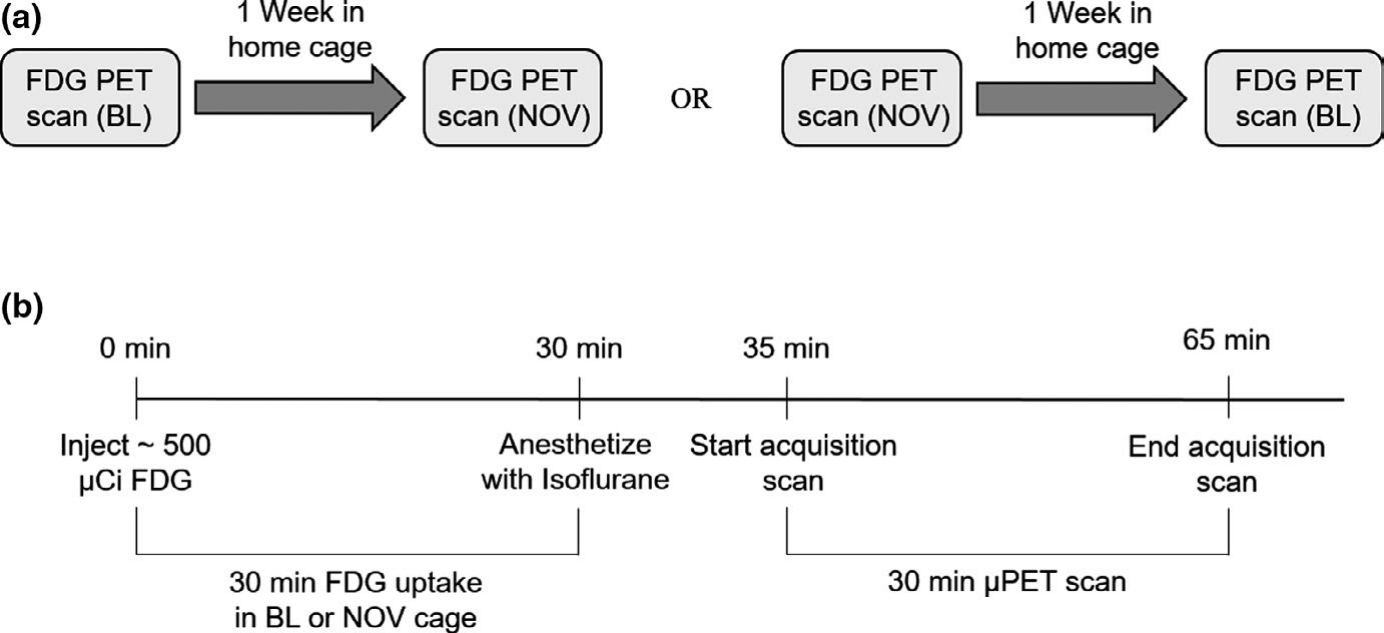
(a), Experimental timeline. Eight animals received the BL scan in their home cage first, followed by the NOV scan in the novel rat cage one week later. The other eight animals received the NOV scan first, followed by the BL scan a week later. Animals remained in their home cage between scans and were not housed near the cage of the novel rat. (b) Timeline of PET procedure. Animals received an intraperitoneal injection of [^18^F] fluorodeoxyglucose (FDG) and were immediately placed in either their home cage (BL) or cage of a novel male rat (NOV) for a 30‐min uptake period. They were anesthetized at the end of the uptake period and placed in the bed of the PET R4 tomograph for the 30‐min scan. Animals were returned to their home cage following recovery

Eight‐week‐old male Sprague Dawley rats (Taconic Farms, Rensselaer, NY) were used in this experiment (*n* = 16). All animals were individually housed in a humidity and temperature‐controlled room (22 ± 2°C and 40%–60% humidity) on a 12‐hr reverse light–dark cycle (lights off 08:00 hr and on at 20:00 hr). Standard laboratory rat chow and water were available ad libitum for the duration of the experiment. Food intake, fluid intake, and body weights were recorded biweekly. PET scans were carried out on each animal twice. Each animal was scanned for 30 min, 30 min after a 500 ± 115 µCi intraperitoneal injection of the radiotracer 18F‐FDG. As a glucose analog, 18F‐FDG is taken up by cells at a rate correlated with cellular activity. The radiotracer remains trapped in the cells for at least 60 min following uptake (Reivich et al., 1979), and so the 18F‐FDG utilization by the awake animal during the uptake period can be measured during the scan of the anesthetized animal. During the 30‐min uptake period, rats were unrestrained and free to move around their environment. The first scan was a baseline (BL) wherein the animal was in its own home cage during 18F‐FDG uptake. The second scan was carried out following 18F‐FDG uptake in the cage of a novel rat (NOV). The other rat was not present during this time. Scans were performed one week apart, with eight rats receiving the NOV scan first and the other eight rats receiving the BL scan first (Figure 1a). Behavioral data were not recorded for analysis. This experiment was conducted in accordance with the National Academy of Sciences Guide for the Care and Use of Laboratory Animals (1996) and approved by the University at Buffalo Institutional Animal Care and Use Committee.

### 2.2 Positron emission tomography imaging

Immediately following the uptake period, rats were anesthetized with 3% isoflurane, maintained on 1% throughout the duration of the scan, and secured on the bed of the scanner. Scans were performed using a PET R4 tomograph (Concorde CTI Siemens), which has a transaxial resolution of 2.0 mm full width at half maximum and a transaxial field of view of 11.5 cm. Scans followed a static imaging protocol for 30 min. Blood glucose levels were measured via tail vein both pre‐ and postscan while the animal was anesthetized (Figure 1b). Animals were food restricted for 8 hr prior to the scan to control for spikes in blood glucose levels. They were monitored until awake, returned to their home cage, and given food and water.

### 2.3 Image and statistical analysis

Images were reconstructed using a MAP algorithm (15 iterations, 0.01 smoothing value, 256 × 256 × 256 resolution), spatially normalized and coregistered to a rat brain MRI template (63 slices) using Paxinos and Watson stereotaxic coordinates with the imaging software PMOD version 2.85 (PMOD Technologies). Statistical Parametric Mapping software (SPM8) was used to identify significant changes in BGluM between BL and NOV scans. A one‐way within subjects ANOVA was performed to identify significant contrasts, with clusters of voxel threshold K > 50 and *p* < .01 set as significant. These clusters were then overlaid onto the rat brain MRI template using AMIDE software (Stanford University). Activation, defined as a statistically significant increase in BGluM in the NOV scan compared to the BL scan, is represented by red/yellow clusters in the figures. Inhibition, defined as greater BGluM in the BL scan than the NOV scan, is represented by blue clusters in the figures.

## 3 RESULTS

**Figure 2:**
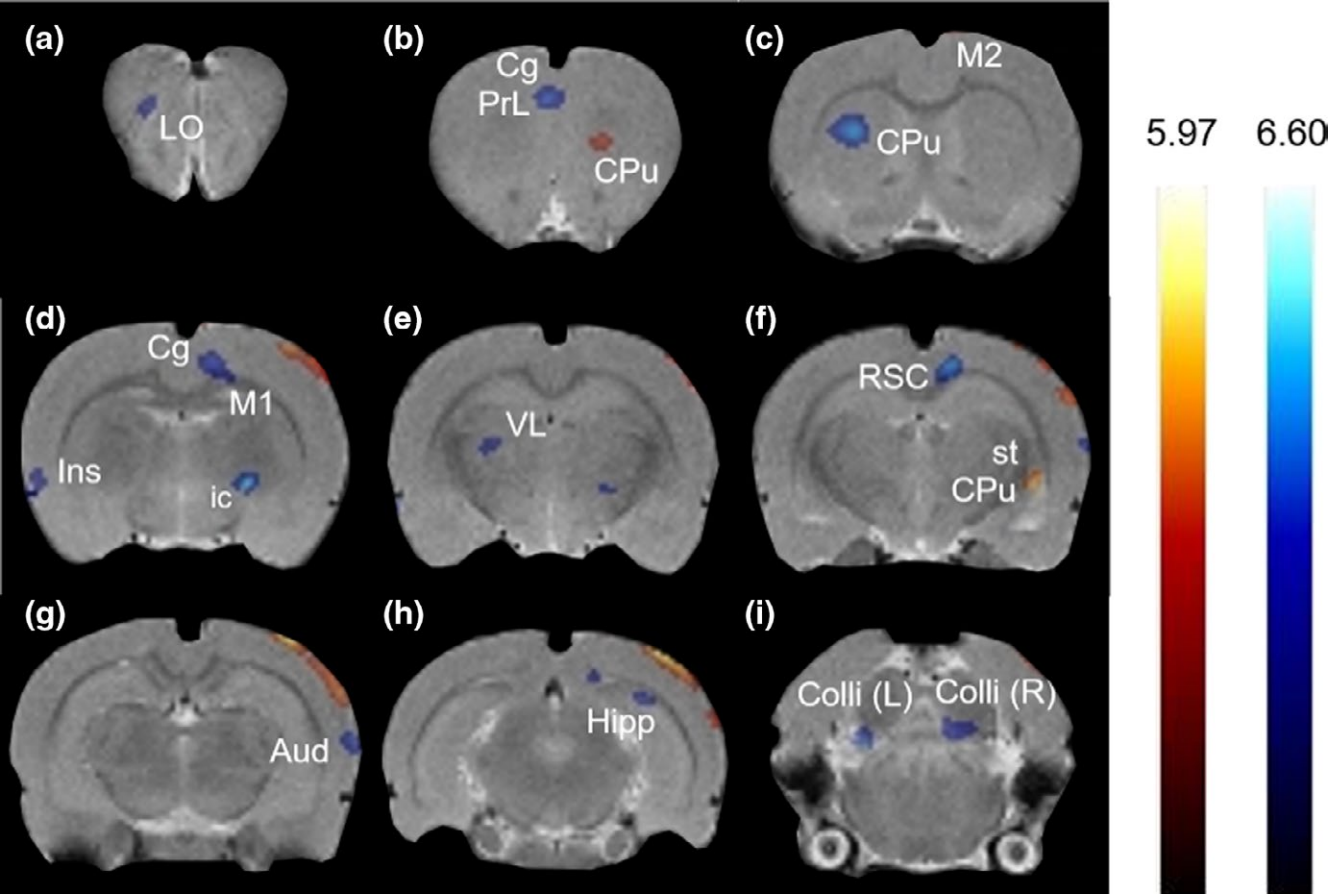
Coronal PET images showing brain regions with significant (*p* < .01, K > 50) differences in brain glucose metabolism (BGluM) between home cage (BL) and novel cage (NOV) scans. Red/yellow clusters illustrate BGluM activation, while blue clusters illustrate BGluM inhibition. (a) Lateral orbital cortex (LO), (b) cingulate cortex (Cg), prelimbic cortex (PrL), striatum (CPu), (c) motor cortex (M2), striatum (CPu), (d) motor cortex (M1, M2), cingulate cortex (Cg), insular cortex (Ins), internal capsule (ic), globus pallidus (GP), (e) ventrolateral thalamus (VL), (f) retrosplenial cortex (RSC), striatum (CPu), stria terminalis (st), (g) auditory cortex (Aud), (h) hippocampus (Hipp), visual cortex (V1B, V2L), and (i) inferior colliculus (Colli)

**Figure 3:**
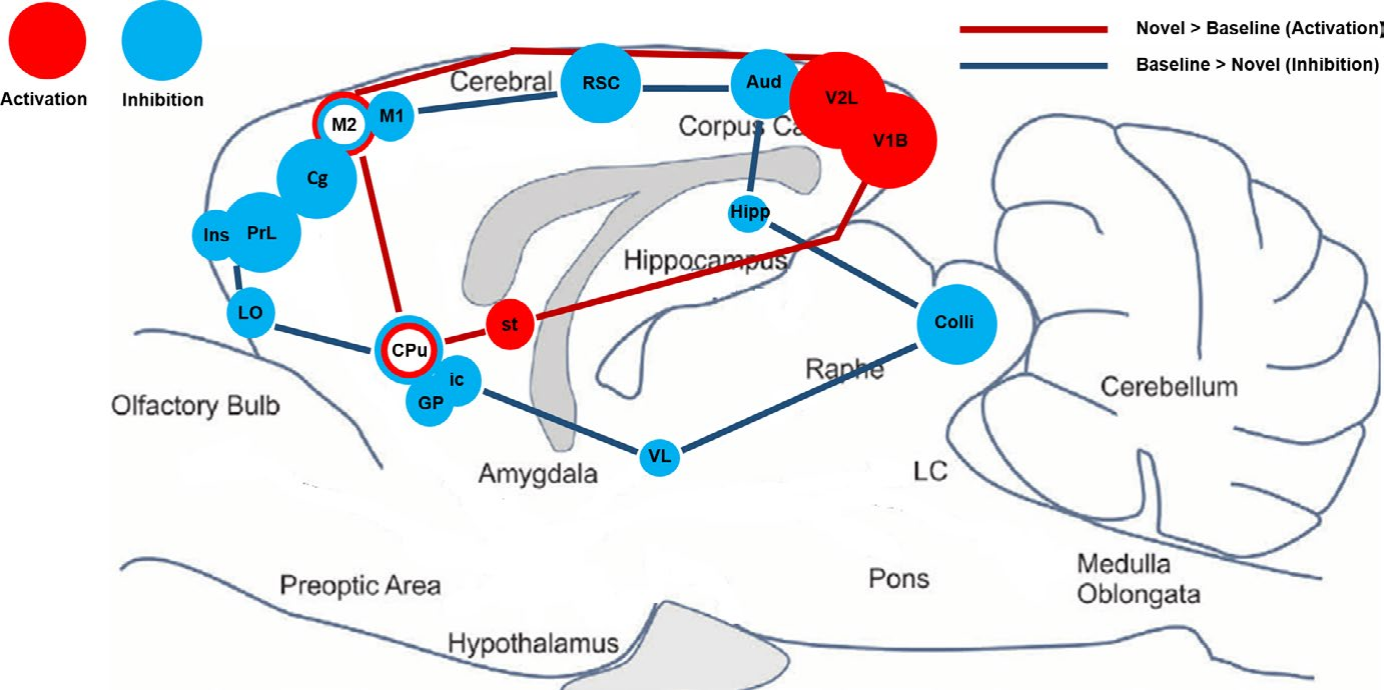
Summary of functional imaging results. Sagittal brain drawing (0.40 mm lateral) showing all regions of statistically significant brain glucose metabolism activation (red) and inhibition (blue) in the rat brain in response to a novel environment. Significant clusters identified for *p* < .01, voxel threshold K > 50. Circle diameter corresponds to cluster size

A one‐way within subjects ANOVA revealed that rats exposed to the novel cage of an unfamiliar male conspecific showed significantly increased (K > 50, *p* < .01) BGluM in the visual cortex, stria terminalis, motor cortex, and the striatum compared to rats in their home cage (NOV > BL; Table 1, Figure 2). BGluM was significantly lower (K > 50, *p* < .01) in the internal capsule, globus pallidus, striatum, retrosplenial cortex, auditory cortex, cingulate cortex, motor cortex, prelimbic cortex, orbitofrontal cortex, hippocampus, insular cortex, inferior colliculus, and ventral thalamic nucleus during uptake in the novel cage, compared to the home cage (NOV < BL; Table 1, Figure 2). These regions of activation and inhibition in response to the novel environment are mapped in the brain as shown in Figure 3.

**Table 1:** Brain regions where there was a significant brain glucose metabolism (BGluM) effect between novel cage (NOV) and home cage (BL) scans at *p* < .01, voxel threshold K > 50

Brain region	Significant effect	Medial–Lateral	Anterior–Posterior	Dorsal–Ventral	t‐value	z‐score	(Ke)
NOV versus BL							
Striatum (CPu)	+	1.8	2.8	4.8	3.35	2.76	70
Secondary motor cortex (M2)	+	0.6	−0.4	0.6	4.14	3.20	136
Stria terminalis (st) Striatum (CPu)	+	4.8	−3.6	6.8	4.56	3.41	114
Primary visual cortex (V1B) Secondary visual cortex, lateral part (V2L)	+	4.6	−7.0	1.2	5.97	3.99	1691
Lateral Orbital cortex (LO)	−	−2.0	5.6	4.0	4.05	3.15	114
Prelimbic cortex (PrL) Cingulate cortex (Cg)	−	−0.4	2.6	3.2	4.22	3.24	339
Striatum (CPu)	−	−3.0	0.2	5.2	4.99	3.60	262
Cingulate Cortex (Cg) Motor Cortex (M1, M2)	−	1.2	−1.4	2.6	4.27	3.27	119
Insular cortex (Ins)	−	−6.4	−1.4	7.0	3.45	2.82	102
Internal capsule (ic) Globus pallidus (GP)	−	2.6	−1.8	7.4	6.60	4.21	118
Thalamus, ventral‐lateral (VL)	−	−2.6	−2.4	5.4	3.25	2.70	86
Retrosplenial cortex (RSC)	−	1.2	−3.8	1.8	4.81	3.52	328
Auditory cortex (Aud)	−	7.0	−4.4	5.0	4.44	3.35	238
Hippocampus (Hipp)	−	3.8	−6.0	3.0	3.55	2.88	78
Inferior colliculus (Colli)	−	−2.8	−8.4	6.0	5.31	3.74	163

Note

Increases (activation) and decreases (inhibition) in BGluM are denoted by ±, respectively. Coordinates in stereotaxic space (Medial–Lateral, Anterior–Posterior, Dorsal–Ventral) are given for the location of the cluster peak. T‐value and z‐score are calculated from the mean BGluM values of all voxels within the significant clusters in the NOV and BL scans. The number of voxels in the significant clusters is given as Ke, voxel size of 0.2 mm isotropic.

## 4 DISCUSSION

Novelty‐seeking behavior is of great interest to cognitive neuroscience researchers due to the associated risks affecting human health. While some have looked at indirect correlates of neural activity in a novel environment, none have used functional imaging to identify the changes in rat brain glucose metabolism that occur. By using PET imaging paired with 18F‐FDG to measure rat BGluM in the novel cage, we have identified brain areas that are possibly activated or inhibited by the novel environment (Figure 3). The roles these regions might play in exploratory and avoidance behavior are discussed.

18F‐FDG uptake while in the cage of the unfamiliar rat resulted in activation of the visual cortex, motor cortex, striatum, and stria terminalis. Activation of the visual cortex in a novel environment is supported by human fMRI studies (Krebs et al., 2009). One group showed a decrease in visual cortex activation following repeated exposure to a stimulus, suggesting the visual cortex is sensitive to novelty. This was associated with a corresponding decrease in amygdala activity, something that we did not see in our study. Perhaps of most interest, decreased activity in these areas was less significant for patients that scored high for anxiety—activation remained higher even with repeated exposure to stimuli (Ousdal, Andreassen, Server, & Jensen, 2014). Our results indicate increased activity of the stria terminalis in the unfamiliar environment, a region associated with anxiety in response to prolonged threats (Hammack, Todd, Kocho‐Schellenberg, & Bouton, 2015). This suggests that the novel (cage) environment may have been a stressor, inducing an anxious state. The corresponding activation of the visual cortex seems to support the idea that attention to novel visual stimuli is boosted by anxiety.

If a novel environment is indeed inducing anxiety in rats, it supports studies that show avoidance behavior in response to a stressful environment (Bevins et al., 2002). Avoidance behavior is characterized by immobility and self‐grooming (Cirulli et al., 1998). Therefore, we should expect to see inhibition in areas associated with movement and exploration, which in fact we did. The striatum (Graybiel & Grafton, 2015), motor cortex (Barthas & Kwan, 2017), and ventrolateral thalamus (Marlinski, Nilaweera, Zelenin, Sirota, & Beloozerova, 2012) are all implicated in the coordination and control of movement, and all were inhibited in the present study in the novel environment. However, both the striatum and motor cortex were also activated at different coordinates of the brain. Without behavioral analysis, we cannot assume the type of behavior that these patterns of activation and inhibition in motor areas correspond to. Notably, the retrosplenial cortex is also inhibited, a region associated with spatial processing and navigation (Mitchell, Czajkowski, Zhang, Jeffery, & Nelson, 2018). This suggests that the rats might be less mobile in the novel cage. It is possible that the motor area activation in this setting may be the result of self‐grooming associated with anxious behavior (Cirulli et al., 1998).

The hippocampus is implicated in spatial navigation, with increased fMRI BOLD activity in a novel environment as compared to a familiar one (Kaplan, Horner, Bandettini, Doeller, & Burgess, 2014). The opposite was true in our study, with the hippocampus being inhibited in the novel environment. However, some studies suggest that the dorsal and ventral subregions of the hippocampus have different functional roles. Lesions to the dorsal hippocampus impair spatial learning but not anxiety, while lesions to the ventral hippocampus reduce anxiety but do not effect spatial learning (Barkus et al., 2010). Animals with lesions to the ventral hippocampus display reduced anxiety as measured by behavior on the elevated plus maze, suggesting that normal ventral hippocampal functioning plays a role in anxiety (Bannerman et al., 2003; Kjelstrup et al., 2002). The inhibition cluster in our results appears in the dorsal hippocampus, meaning this subregion is more active in the rat's home cage. This also supports the idea that the animal is navigating more in its home cage, requiring the activation of spatial memory. However, we do not see the significant activation in the ventral hippocampus we might expect if the animal were more anxious in the novel environment.

A significant inhibition cluster encompassed the globus pallidus and internal capsule in the unfamiliar environment. The globus pallidus has been implicated in the control of movement through projections to the thalamus (Goldberg, Farries, & Fee, 2013; Lanciego, Luquin, & Obeso, 2012). The internal capsule is composed of white matter, a bundle of axons that carries information to the cerebral cortex from various other areas of the brain. Lesions to the internal capsule cause motor impairment, suggesting a role in motor control as well (Lee et al., 2000). White matter activation is rarely looked at in FDG‐PET imaging, because its glucose consumption is 2.5 to 4.1 times less than gray matter; however, it is possible and has been done (Jeong, Yoon, & Kang, 2017). These regions of inhibition indicate that brain motor functions are suppressed in the unfamiliar environment, further supporting the assumption that rats are less mobile in the novel cage.

Auditory processing areas were also inhibited in the novel cage. fMRI studies have shown that spontaneous (in the absence of auditory stimuli) activation of the auditory cortex is accompanied by activity in the anterior cingulate cortex (Hunter et al., 2006). Both of these areas were inhibited in the unfamiliar environment. The insular cortex was also inhibited, a region associated with audiovisual sensory integration (Bushara, Grafman, & Hallett, 2001). This is the opposite of human fMRI results that show insular and temporal cortex activation in response to novelty (Ranganath & Rainer, 2003).

The cingulate cortex plays a role in memory and decision making and is thought to form knowledge of the environment based on past experiences output from hippocampal memory (Mashhoori, Hashemnia, McNaughton, Euston, & Gruber, 2018). Its activity also correlates with sustained attention (Wu et al., 2017). This area was inhibited in the unfamiliar environment, the opposite of the results of human fMRI studies that showed cingulate activation in response to a novel cue (Krebs et al., 2009; Ranganath & Rainer, 2003). Similarly, the orbitofrontal cortex integrates current sensory input with prior experience to inform decision making—this area was inhibited in the novel environment of our study (Furuyashiki, Holland, & Gallagher, 2008; Nogueira et al., 2017).

The prelimbic cortex is involved in working memory (Gisquet‐Verrier & Delatour, 2006). Lesions to the prelimbic cortex have also been shown to increase anxiety, as measured by activity in the elevated plus maze (Jinks & McGregor, 1997). Therefore, we would expect increased prelimbic cortex activity to correspond to increased anxiety. However, this region was inhibited in the novel environment in this study, challenging the assumption that the unfamiliar cage induced anxiety in rats. Perhaps the decreased activity is an inhibition of working memory, which might be less active if the rat was exploring less in the novel environment.

The orbital, prelimbic, retrosplenial, cingulate, auditory, visual, and postparietal cortices, along with the dorsal hippocampus, have all been described as being part of the default mode network (DMN) of the rat brain (Hsu et al., 2016; Lu et al., 2012). The DMN in humans is associated with restfulness and introspection and is deactivated during cognitive tasks and external directed attention (Raichle et al., 2001). Interestingly, all these regions except the postparietal and visual cortices were inhibited in the novel environment of our study. This DMN inhibition is indicative of working memory (Anticevic, Repovs, Shulman, & Barch, 2010) and has been supported by rat fMRI studies showing similar network inhibition when exposed to an unfamiliar testing environment (Upadhyay et al., 2011). We propose that this DMN inhibition is indicative of heightened attention to the environment in the novel cage. This is in contrast to the rat's home cage, where the DMN is activated. Also of note, local field potential gamma band activity in DMN‐associated regions, which increases in humans during DMN‐related behaviors (Dastjerdi et al., 2011; Ossandon et al., 2011; Ramot et al., 2012) and correlates with cortical blood oxygen level‐dependent (BOLD) activation (Logothetis, 2003), is negatively correlated with exploratory behavior and positively correlated with self‐grooming and quiet wakefulness in rats (Nair et al., 2018). Without behavioral data, we cannot confirm this correlation in our study.

The patterns of activation and inhibition seen in this study are similar to those reported in other functional imaging studies during exposure to a stressful cue. An FDG‐PET study of rats subjected to acute stress (1 hr immobilization) showed significant deactivation in the dorsal hippocampus, thalamus, motor and somatosensory cortices, and striatum, regions that were also inhibited in our study when rats were exposed to the unfamiliar environment (Sung et al., 2009). Significant inhibition was also seen in the cerebellum and superior colliculus (no significance in our study) and visual cortex (activated in our study), with significant increases in BGluM in the hypothalamus, entorhinal, and insular/piriform cortices (no significance in our study). Humans exposed to a psychosocial stressor show deactivation in the hippocampus and medial orbitofrontal and anterior cingulate cortices, regions that were also inhibited in our study (Pruessner et al., 2008). This further supports our hypothesis that the novel cage is a stress‐inducing environment.

It should be noted that stress levels in a novel environment are lower compared to such stresses as chronic restraint. Previous studies have shown that restrained rats have plasma corticosterone concentrations around 30 µg/dl (Marin, Cruz, & Planeta, 2007), whereas levels in a novel environment are closer to 20 µg/dl (Terranova et al., 1999). These levels drop to below 15 µg/dl when the rat is placed in a novel environment with a familiar or unfamiliar conspecific. Baseline concentration is typically below 1 µg/dl. So regardless of the potentially stress‐attenuating effects of the olfactory cues present in the novel male rat cage, we would still expect to see elevated stress levels compared to the home cage. The patterns of activation in the novel environment in this study suggest induced stress, but we cannot comment on the degree to which this mild stress is attenuated by the olfactory cues of the unfamiliar male conspecific.

## 5 CONCLUSIONS

Our results show an increase in BGluM in regions associated with visual processing, motor function, and anxiety in a novel environment, with decreased activity in auditory processing, locomotor function, spatial navigation, and working memory regions, including regions of the DMN. These patterns of inhibition suggest decreased exploration in the novel environment, a surprising result given rat preference for a novel environment in previous behavioral studies. Activation of anxiety‐associated regions indicates that the novel cage may be a stressful environment, which would explain a decreased exploratory drive. Future studies could determine how these patterns of activation would change in a clean novel environment, without the potentially stress‐attenuating presence of stimuli associated with another rat. Behavioral analysis is also needed to correlate BGluM with specific exploratory or avoidance behaviors.

Mapping BGluM in response to a novel environment is important when attempting to understand the cognitive and behavioral changes that are induced in rodents when exposed to novel experimental setups. Researchers should habituate animals to the experimental environment prior to testing whenever possible, to avoid inducing a stress response and impacting behavior. Decreased exploration must be expected and accounted for where habituation is not possible. While we cannot distinguish the effects of the olfactory cues of the novel rat and the novel environment itself in this experiment, both may contribute to the changes in brain activity described. Particularly in PET studies, 18F‐FDG uptake should occur in the animal's home cage to minimize unwanted changes in brain activity. These methods must be considered when interpreting the results of brain imaging studies. In addition, these data demonstrate the potential of FDG‐PET imaging to examine brain activation in response to novelty in the context of decision making and risk taking, along with the cognitive effects of stressful stimuli. This presents the opportunity to examine cognitive differences in novelty‐seeking subjects that are vulnerable to risky behaviors. When combined with behavioral neuroscience experiments, this brain imaging approach could help provide a functional map of brain regions and circuits in response to psychoactive drugs, stressors, or cues involved in learning.

## CONFLICT OF INTEREST

All authors do not have any conflict of interest to disclose.

## AUTHORS CONTRIBUTION

MM and PKT prepared the manuscript; MM, KR, and PT analyzed the data; KR and MA involved in the animal work.

## Data Availability

The data supporting the findings of this study are available from the corresponding author upon reasonable request.
